# Adult-specific collagen COL-19 is dispensable for contact-mediated mate recognition in *Caenorhabditis elegans*

**DOI:** 10.17912/micropub.biology.001141

**Published:** 2024-02-22

**Authors:** Jen-Wei Weng, Chun-Hao Chen

**Affiliations:** 1 Institute of Molecular and Cellular Biology, National Taiwan University, Taipei, Taiwan

## Abstract

Mate recognition in *C. elegans* involves the integration of multiple sensory cues to facilitate the identification of suitable mates for reproductive behaviors. The cuticle, serving as the protective outer layer enveloping the entire body, has been implicated in eliciting contact responses essential for contact-mediated mate recognition in males. However, the specific constituents of cuticular cues have yet to be identified. In this study, we investigate the potential modulatory role of adult-specific collagen COL-19 in contact-mediated mate recognition. Our study shows that the expression of COL-19 ::GFP is adult-specific and not sexually dimorphic. Knockdown of *col-19* via RNAi does not affect mate attractiveness of hermaphrodites in male retention assay, as corroborated by generating two independent *col-19* putative null mutants via CRISPR/Cas9. These findings suggest that *col-19* does not contribute to contact-mediated mate recognition, thereby advancing our mechanistic understanding of the intricate social interactions between sexes in *C. elegans* .

**Figure 1. Characterization the role of col-19 in contact-mediated mate recognition:**
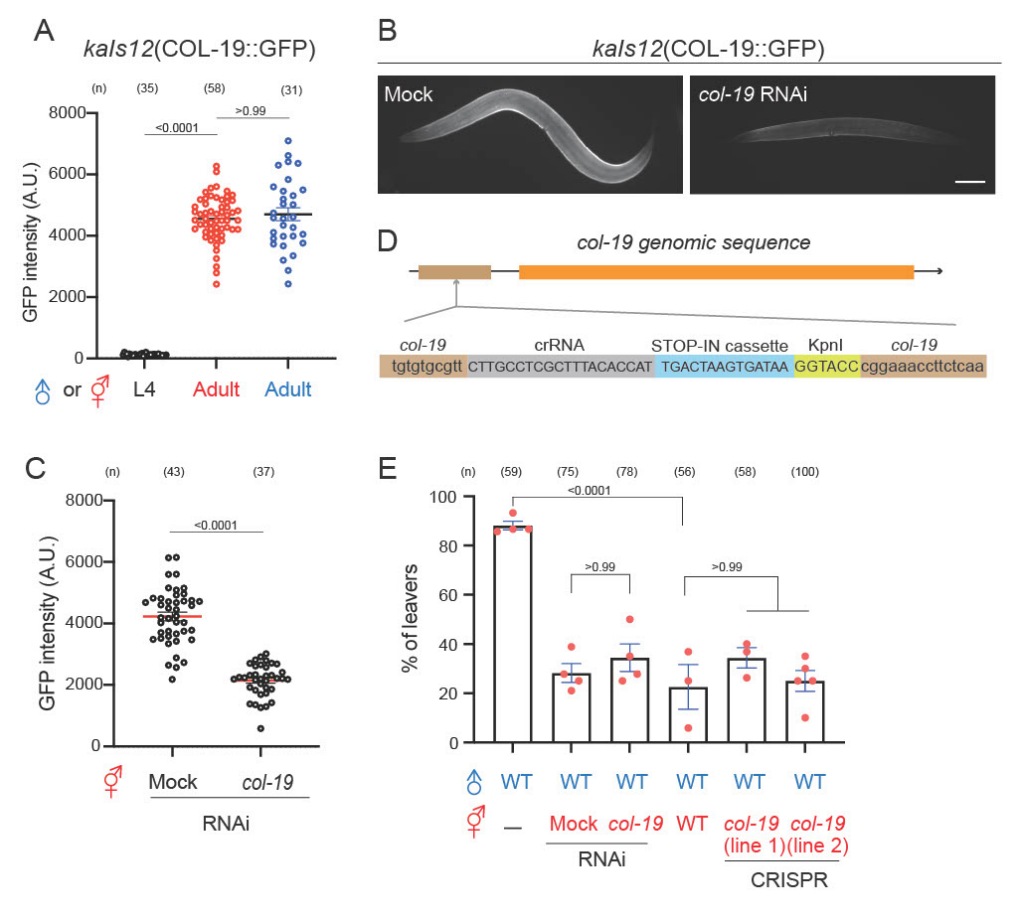
(A) Quantification of COL-19 ::GFP *( kaIs12 )* fluorescent signal with animals at L4 stages and adults. One-way ANOVA with Bonferroni correction and *p* values indicated. (B) Images of COL-19 ::GFP hermaphrodites treated by RNAi. Scale bar = 100 μm. (C) Quantification of the fluorescence intensity of COL-19 ::GFP hermaphrodites treated by RNAi. t test with *p* values indicated. (D) Genomic sequences of *col-19* putative null mutants generated by CRISPR/Cas9 STOP-IN method. (E) Percentage of male leavers in the male retention assay. The dots represent 15-20 males in one replica. Error bar = S.E.M. One-way ANOVA with Bonferroni correction for statistical comparison and *p* values indicated.

## Description

Mating behavior is important for animals to produce reproductive offspring. To recognize suitable mates, animals rely on multiple sensory cues to locate potential mates (Billeter et al., 2006; Liberles, 2014; Toda et al., 2012) . In *C. elegans* , long-range cues such as ascarosides and volatile pheromones produced by hermaphrodites attract males remotely (Edison, 2009; Simon & Sternberg, 2002; Wan et al., 2019) . Upon close proximity, physical contact becomes pivotal for transmitting multiple sensory cues, leading to contact-mediated mate recognition (Barrios et al., 2008; Lipton et al., 2004; Weng et al., 2023) . Previously, our lab revealed that contact-mediated mate recognition requires a two-step sensory mechanism. Initially, cuticular cues on the surface of hermaphrodites elicit contact responses of males to guide the selection of conspecific and reproductive mates, followed by a recognition of specific body stiffness to validate the suitability of mates. These two sensory cues are essential for males to recognize and stay with suitable mates (Weng et al., 2023) . Although cuticular cues play a crucial role, the surface molecules constituting cuticular cues remain unidentified.

*C. elegans* cuticles play a role in body patterning and function as the primary barrier to prevent infection by pathogens (Page & Johnstone, 2007) . The cuticles, mainly composed of collagens, are synthesized by hypodermis to shield the whole body (Page & Johnstone, 2007) . During molting, worms shed the old cuticle and synthesize various collagens, including furrow collagens such as *dpy-2* , *dpy-7* , and *dpy-10* (Johnstone & Barry, 1996; McMahon et al., 2003; Page & Johnstone, 2007) . Our previous findings suggest that these collagens contribute to body stiffness for contact-mediated mate recognition regarding sexual identity and reproductive status (Weng et al., 2023) . While these results suggest a role for cuticular collagens in social interactions via mechanical signaling, whether cuticular collagens play versatile roles in contact-mediated mate recognition is unknown.

COL-19 is an adult-specific collagen on the acme of the annulus that has been used as a genetic marker to visualize the surface of cuticles (Thein et al., 2003) . Given the stage-specific expression profile, we hypothesized that adult-specific collagen, COL-19 , may function as a cuticular cue for mate recognition. Through visualization of COL-19 protein tagged with GFP within an integrated transgenic strain *kaIs12* , we confirmed that COL-19 was expressed in adult but not L4 larvae hermaphrodites ( Fig. 1A ). The expression level of COL-19 ::GFP was comparable in hermaphrodites and males, suggesting that *col-19* is not sexually dimorphic ( Fig. 1A ). We next performed *col-19* feeding RNAi and showed that COL-19 ::GFP intensity was significantly reduced ( Fig. 1B and 1C). However, the reduction of *col-19* activity in hermaphrodites by RNAi did not affect the retention of males, suggesting that mate recognition is not affected ( Fig. 1E ). Since residual *col-19* activity treated by RNAi might still contribute to mate recognition, we created putative *col-19* null mutants by inserting a STOP-IN cassette through the CRISPR-Cas9 method (Wang et al., 2018) ( Fig. 1D ). Similar to the *col-19* RNAi, two independent CRISPR-edited strains effectively retained males as wild-type hermaphrodites ( Fig. 1E ). It is noteworthy that male retention assay may not be sensitive enough to uncover potential role of *col-19* in mate recognition controlled by long-range and short-range cues. Future experiments, such as recently developed mate choice assay (Luo et al., 2023) , are needed to investigate the possibility. Altogether, these data indicate that *col-19* is dispensable in contact-mediated mate recognition, expanding our understanding of the intricate mechanisms governing social interactions in *C. elegans* .

## Methods

**Strains**

*C. elegans* strains were cultured and maintained on nematode growth medium (NGM) plates seeded with *E. coli* OP50 bacteria at 20°C (Brenner, 1974) . The alleles of the worms used in this study are listed in Reagent Table.

**Male Retention Assay**

The male retention assay was conducted as previously described (Barrios et al., 2008) . The assay was performed in a 9 cm plate with 10 mL nematode growth medium (NGM) agar. Each plate was seeded with 18 μL OP50 bacteria (OD600=0.4-0.6) in the center a day before the assay. Before the assay, D1 adult hermaphrodites (mates) were fixed with 8% paraformaldehyde for 30 min at room temperature, and then fixed worms were washed with M9 buffer. Next, one virgin D1 male was placed with or without two mates on the food patch of each assay plate. One experimental set contains 15-20 virgin D1 males. A male is considered a leaver when the moving track reaches the set boundary (0.5 cm away from the plate edge). Plates were kept at 20°C and the proportion of males was scored after 24 hours. The percentage of leaver is calculated by dividing the number of leavers by the number of total tested animals, as shown below.

% of leavers = (# of leavers / # of total animals) x 100

The value of the percentage of leaving males is pooled across replicas to calculate the standard error of the mean (S.E.M.).

**RNA Interference**

Feeding RNAi experiments were conducted as previously described (Kamath et al., 2000) . 1 kb complementary DNA (cDNA) sequences of *col-19* excluding the start codon were amplified from a home-made cDNA library and were cloned into the L4440 plasmid using the primers: *col-19* forward primer (5′-GCAAGCTCATTGTGGTTGGAT-3′) and *col-19* reverse primer (5′-CTCGTGCAGCTTACAAGGCT-3′). Plasmids were transformed into *E. coli* HT115 bacterial strain as a food source for feeding RNAi experiments. For the preparation of RNAi bacteria, a single colony was cultivated in LB overnight. Then culture bacteria were refreshed by diluting 1:100 to fresh LB and incubated at 37 °C for 3-4 hours until the OD600 reached 0.4-0.6, followed by RNAi production with 1 mM isopropyl β-D-1-thiogalactopyranoside (IPTG) for 1 hour at room temperature. NGM plates with 1 mM IPTG and 50 μg/mL ampicillin were seeded with 100 μL bacterial culture. Worms were synchronized and arrested at L1 (P0) larvae and then reared on RNAi plates for two generations. Quantification was done with F2 animals.

**Microinjection**

Germline transformation was performed by microinjecting plasmids in gonads as described (Mello et al., 1991). An inverted microscope (Zeiss, Axio Observer) was used to visualize the gonads of D1 adult worms, and the crRNA/DNA/Cas9 mixture with co-injection markers was injected through a microinjector (Eppendorf FemtoJet 4X).

**CRISPR/Cas9 Mutagenesis**

CRISPR-Cas9 stop-in method was employed as described with modifications (Wang et al., 2018) . IDT website (IDT, Coralville, IA) was used to design the crRNA and the tracrRNA. The guide RNA sequences for all deletion mutants generated by the CRISPR STOP-IN method were listed in the reagent Table. In brief, 0.5 μL of tracrRNA (0.4 μg/μL), 0.5 μL of crRNA (0.4 μg/μL), 0.5 μL of the Cas9 protein (from IDT) mixed with 6.8 μL RNAase-free water were incubated at 37 °C for 10 minutes. After cooling down at room temperature, 0.5 μL DNA donor (1 μg/μL) , 40 ng pRF4( *rol-6 ( su1006 )* ), and RNase-free water were then added to the mixture to reach a final volume of 20 μL. The CRISPR-Cas9 complex was used for microinjection with N2 *(C. elegans).* Picked the worms with Rol phenotype. The sequence was amplified by PCR and digested with KpnI enzyme for genotyping.

**Microscopy and Quantification of Images**

For the microscopy experiment, worms were mounted on 2 % agarose pads and anesthetized with 1 % sodium azide. Samples were observed under Apotome (Zeiss ApoTome2) and M2 Zeiss Imager Z2 microscope with an Axiocam 506 Mono camera and processed by using ZEN Blue 3.3 software, and the fluorescence intensity was quantified by ImageJ software.

**Quantification and statistical analysis**

One-way ANOVA with Bonferroni correction and t test was conducted by Prism (Version 9.5.1) as indicated in Figure Legends. Error bars in bar graphs represent the standard error of means (S.E.M.).

## Reagents

**Table:** 

Strain	Genotype	Available from
N2	*C. elegans*	CGC
TP12	*kaIs12 (col-19::GFP) *	CGC
NTU83	*col-19 ( chc3 ) *	This study
NTU84	*col-19 ( chc4 ) *	This study

Bacteria strain	Genotype	Available from
*E. coli*	OP50	CGC
*E. coli*	HT115	Yi-Chun Wu's Lab

Chemicals	Source	Description
Paraformaldehyde	Agar Scientific	Catalog number: AGR1026

## References

[R1] Barrios Arantza, Nurrish Stephen, Emmons Scott W. (2008). Sensory Regulation of C. elegans Male Mate-Searching Behavior. Current Biology.

[R2] Billeter JC, Rideout EJ, Dornan AJ, Goodwin SF (2006). Control of male sexual behavior in Drosophila by the sex determination pathway.. Curr Biol.

[R3] Brenner S (1974). The genetics of Caenorhabditis elegans.. Genetics.

[R4] Edison AS (2009). Caenorhabditis elegans pheromones regulate multiple complex behaviors.. Curr Opin Neurobiol.

[R5] Johnstone IL, Barry JD (1996). Temporal reiteration of a precise gene expression pattern during nematode development.. EMBO J.

[R6] Kamath RS, Martinez-Campos M, Zipperlen P, Fraser AG, Ahringer J (2000). Effectiveness of specific RNA-mediated interference through ingested double-stranded RNA in Caenorhabditis elegans.. Genome Biol.

[R7] Liberles SD (2013). Mammalian pheromones.. Annu Rev Physiol.

[R8] Lipton J, Kleemann G, Ghosh R, Lints R, Emmons SW (2004). Mate searching in Caenorhabditis elegans: a genetic model for sex drive in a simple invertebrate.. J Neurosci.

[R9] Luo J, Barrios A, Portman DS (2023). C. elegans males optimize mate-choice decisions via sex-specific responses to multimodal sensory cues.. bioRxiv.

[R10] McMahon L, Muriel JM, Roberts B, Quinn M, Johnstone IL (2003). Two sets of interacting collagens form functionally distinct substructures within a Caenorhabditis elegans extracellular matrix.. Mol Biol Cell.

[R11] Page AP, Johnstone IL (2007). The cuticle.. WormBook.

[R12] Simon JM, Sternberg PW (2002). Evidence of a mate-finding cue in the hermaphrodite nematode Caenorhabditis elegans.. Proc Natl Acad Sci U S A.

[R13] Thein MC, McCormack G, Winter AD, Johnstone IL, Shoemaker CB, Page AP (2003). Caenorhabditis elegans exoskeleton collagen COL-19: an adult-specific marker for collagen modification and assembly, and the analysis of organismal morphology.. Dev Dyn.

[R14] Toda H, Zhao X, Dickson BJ (2012). The Drosophila female aphrodisiac pheromone activates ppk23(+) sensory neurons to elicit male courtship behavior.. Cell Rep.

[R15] Wan X, Zhou Y, Chan CM, Yang H, Yeung C, Chow KL (2019). SRD-1 in AWA neurons is the receptor for female volatile sex pheromones in C. elegans males.. EMBO Rep.

[R16] Wang H, Park H, Liu J, Sternberg PW (2018). An Efficient Genome Editing Strategy To Generate Putative Null Mutants in
*Caenorhabditis elegans*
Using CRISPR/Cas9.. G3 (Bethesda).

[R17] Weng JW, Park H, Valotteau C, Chen RT, Essmann CL, Pujol N, Sternberg PW, Chen CH (2023). Body stiffness is a mechanical property that facilitates contact-mediated mate recognition in Caenorhabditis elegans.. Curr Biol.

